# The efficacy and safety of auricular acupuncture versus electroacupuncture in ameliorating chemotherapy-induced nausea and vomiting among patients receiving cisplatin-based regimens

**DOI:** 10.1097/MD.0000000000024588

**Published:** 2021-02-19

**Authors:** Meng-yue Liu, Lai Sung, Yan-Sen Liao, Yi-fei Jiao, Chang-sheng Sun, Xiao-dong Peng

**Affiliations:** a Department of Traditional Chinese Medicine; b Oncology Department, Chengdu Second People's Hospital; c Department of Traditional Chinese Medicine, Chengdu Seventh People's Hospital, Chengdu, Sichuan Province, P.R. China.

**Keywords:** auricular acupuncture, chemotherapy-induced nausea and vomiting, cisplatin-based regimen, electroacupuncture

## Abstract

**Background:**

Nausea and vomiting are among the most common and distressing side effects of chemotherapy. Difference in views about the effectiveness of auricular acupuncture (AA) versus electroacupuncture (EA) of chemotherapy-induced nausea and vomiting (CINV) lies at the heart of the debate. The aim of this study is to compare the antiemetic efficacy and safety of AA and EA for CINV.

**Methods:**

One hundred twenty participants, 18 to 75 years old malignant tumors will receiving chemotherapy with cisplatin, will be recruited and randomized into 3 groups equally, Group A (the AA group), Group B (the EA group), and Group C (the control group). The participants in Group A and Group B will receive AA or EA regimens, alternatively, beginning on the day before first day of chemotherapy for a third consecutive cycles. All participants will continue to receive conventional treatment. The incidence and severity of CINV will be assessed using the definition and classification of nausea and vomiting (NCI-CTC AE4.0) and the MASCC (Multinational Association for Supportive Care in Cancer) Antiemesis Tool (MAT). Secondary outcome measures include the degree of abdominal distension, the first time of flatus and defecation, and life quality. Additionally, adverse events will also be documented during the period of the treatment.

**Discussion:**

This trial may provide evidence regarding the clinical effectiveness and safety of AA versus EA for CINV following cisplatin-based regimens.

**Trail registration:**

This study is registered with the Chinese Clinical Trial Registry: ChiCTR2000040942.

## 1 Introduction

Chemotherapy-induced nausea and vomiting (CINV) is among the most common and distressing side effects of chemotherapy.^[1]^ It has been shown to significantly affect the quality of life (QoL) and daily function of patients receiving chemotherapy.^[2]^ Moreover, CINV, as a prognostic factor for overall survival, is so severe in some cases that it interrupts the treatment of primary cancer and increases the risk of disease progression,^[3,4]^ which result in treatment-outcome reduction.^[5]^

Cisplatin, which is a cornerstone of chemotherapy for the treatment of multiple cancers, is a highly emetogenic chemotherapy drug.^[6]^ Over 90% cancer patients will vomit within the first 24 hours after the administration of cisplatin-based chemotherapy unless antiemetic drugs are used.^[7]^ Even with the guideline-recommended antiemetic drugs, cisplatin-induced vomiting is controlled in only 60% of patients; therefore, considerable numbers of patients still experience nausea.^[8]^

Although the mechanisms underlying CINV are not clear enough,^[9]^ to date, commonly prescribed medicines,^[10]^ for nausea and vomiting are: 5-hydroxytryptamine-3 serotonin receptor antagonists (5-HT3RA), Neurokinin1 receptor antagonists (NK-1RA), and corticosteroid.^[11]^ However, for all its prominence in CINV management, these drugs are subject to considerably economic burden and side effects, namely insomnia, headaches, dizziness, and constipation.^[12]^ Thus, additional treatments are urgently needed to effectively manage and ameliorate chemotherapy-induced nausea and vomiting (CINV), without those defects in the wake of antiemetic drugs.

An increasing body of evidence points that electroacupuncture (EA) may provide modest benefits in the treatment of CINV.^[13]^ However, the spread of it is surprisingly slow, and EA is still judged as a highly suggestive but not conclusive treatment.^[14–17]^ For certain patients, they are reluctant to accept EA treatment for their intolerance to needling. The problems and limits of EA center on how it manipulate. Thus, auricular acupuncture (AA) might be a sound treatment to feather all patients’ needs for convenience and comfort. Compared with a pile of studies conducted the access to the effectiveness of EA against CINV, the role of AA has received less attention,^[18]^ and the literatures on AA are limited in number with uncertain quality.

To our knowledge, there are no randomized controlled trails comparing the effect and safety of AA versus EA in cancer patients who experience CINV. Therefore, the present study is designed to determine the efficacy and safety of AA versus EA in ameliorating CINV among patients receiving cisplatin-based regimens.

## 2 Methods

### 2.1 Study design

This is a singular center, randomized, placebo-controlled clinical trial. Using a balanced random approach, all participants will be recruited and assigned to 3 groups: Group A (the AA group), Group B (the EA group), and Group C (the control group), after meeting the eligibility criteria, and completing informed consent.

### 2.2 Ethics

This trial will be accomplished strictly followed the principles for medical ethics outlined in the 2013 updated Declaration of Helsinki. The study was approved by the Ethics and Research Committee of Chengdu Second People's Hospital, China (ethics reference: 2020077). This committee is an ethical approval committee organized by leading professors in their own fields. It will be obtained from all participants in advance that written and signed informed consent. This trial has been registered on www.chictr.org.cn/edit.aspx?pid=63281&htm=4 (ChiCTR2000040942) at December 16, 2020 and will be reported squared with the CONSORT statement,^[19]^ as well as STRICTA (Standards for Reporting Interventions in Clinical Trials of Acupuncture).^[20]^ The Chinese Cochrane Center is responsible for the whole trial including the randomization, blinding, statistical analyses, data management, and monitoring.

### 2.3 Population

All participants will be recruited from the Oncology Department of Chengdu Second People's Hospital, a tertiary hospital, which plays the leading role at the local. We will recruit cases diagnosed with magnificent tumor confirmed by clinicopathology or cytology undergoing the cisplatin-based chemotherapy regimens. Participants who fulfil the inclusion criteria and sign informed consent forms will enter the screening period, otherwise will be excluded before randomization. Chengdu is the largest base of industrial technology and commercial and financial center in the west of China, with a population of 14,047,600, where could collect more cases of diversity. The duration of enrollment is from December 31, 2020 to December 30, 2022.

Eligible participants are all adults aged 18 to 75 years, diagnosed with magnificent tumor confirmed by clinicopathology or cytology undergoing the cisplatin-based chemotherapy regimens and an expected survival time of >3 months. They are still in relatively good physical condition, with a Kamofsky score >60.

The exclusion criteria are as follows: nausea and vomiting caused by intracranial or other metastases, severe gastrointestinal obstruction, or combine organic disorders; recent using drugs may cause nausea and vomiting; participants with allergy history, especially to metal and tape; participants with inability to communicate; participants participating in another clinical trial within 3 months.

The withdrawal criteria are as follows: terminate or adjust the chemotherapy regimen after evaluation by oncologists due to clinical exacerbation; at the patient's own request or at the request of their legal representative.

### 2.4 Intervention

Participants in all groups will receive conventional Western medical treatments as recommended by the Guidelines of Chinese Society of Clinical Oncology: Prevention and treatment of nausea and vomiting caused by antitumor therapies,^[21]^ including 5-HT3 receptor antagonist, NK-1 receptor antagonist, dexamethasone, and so on. In addition to conventional treatment, patients in Group A and Group B will receive AA or EA regimens, alternatively.

Patients receiving acupuncture therapy will be treated bilaterally at 2 distal acupoints: Neiguan (PC6) and Zusanli (ST36). PC6 is an acupoint of the pericardium meridian. It is located between the palmaris longus tendon and the flexor carpi radialis muscle tendon, 2 cun above the rasceta. ST36 is located on the anterolateral side of the lower leg, 3 cun below Dubi and one finger width lateral of the anterior border of the tibia. The location of these acupoints is described in The National Standard of Acupoint Location. Detailed information on the acupuncture treatment is provided in Table 1.

**Table 1:** Detailed information about acupuncture treatment according to STRICTA guideline.

Item	Detail	Detail response
1. Acupuncture rationale	(1a) Style of acupuncture (e.g., Traditional Chinese Medicine, Japanese, Korean, Western medical, five element, ear acupuncture, etc)	Group A: Auricular acupuncture, also named as ear acupunctureGroup B: Electroacupuncture
	(1b) Reasoning for treatment provided, based on historical context, literature sources, and/or consensus methods, with references where appropriate	Selected acupoints based on literature review and clinical experience
	(1c) Extent to which treatment was varied	No variation
2. Details of needling	(2a) Number of needle insertions per subject per session (mean and range where relevant)	4
	(2b) Names (or location if no standard name) of points used (uni/bilateral)	Neiguan (PC6) and Zusanli (ST36)Bilaterally used
	(2c) Depth of insertion, based on a specified unit of measurement, or on a particular tissue level	Group A: 1.2 mmGroup B: 1–2 cm
	(2d) Response sought (e.g., *de qi* or muscle twitch response)	Group A: PainGroup B: *de qi*
	(2e) Needle stimulation (e.g., manual, electrical)	Group A: NoneGroup B: Electrical
	(2f) Needle retention time	Group A: 1 dayGroup B: 30 min
	(2g) Needle type (diameter, length, and manufacturer or material)	Group A: Sterile intradermal acupuncture needles (PYONEX, 0.21.2 mm, SEIRIN Corporation, Japan)Group B: Sterile single-use needles (Shukang, ϕ0.25 × 40 mm, Changchun Aikang Medical Devices Co., Ltd., China)
3. Treatment regimen	(3a) Number of treatment sessions	Three consecutive chemotherapy cycles
	(3b) Frequency and duration of treatment sessions	Once a day
4. Other components of treatment	(4a) Details of other interventions administered to the acupuncture group (e.g., moxibustion, cupping, herbs, exercises, lifestyle advice)	None
	(4b) Setting and context of treatment, including instructions to practitioners, and information and explanations to patients	The same practitioner will treat every session for 1 participant.
5. Practitioner background	(5) Description of participating acupuncturists (qualification or professional affiliation, years in acupuncture practice, other relevant experience)	Licensed Traditional Chinese medicine doctor with >5 years of acupuncture treatment experience
6. Control or comparator interventions	(6a) Rationale for the control or comparator in the context of the research question, with sources that justify this choice	No acupuncture will be performed on the control group
	(6b) Precise description of the control or comparator. If sham acupuncture or any other type of acupuncture-like control is used, provide details as for Items 1 to 3 above.	No sham acupuncture will be performed on the control group

Participants in Group A will receive the following treatment. After each insertion site be disinfected with 75% alcohol, sterile intradermal acupuncture needles (PYONEX, ϕ0.2 × 1.2 mm, SEIRIN Corporation, Japan) will be inserted vertically to a depth of 1.2 mm. Participants will be requested to put pressure on the each auricular point for about 15 seconds per one time, 3 times per day.

As for the participants in Group B, Shukang brand needles (size 0.25 mm × 40 mm) made by Changchun Aikang Medical Devices Co., Ltd., China, will be used to perform the acupuncture treatments. Stimulation will be applied until the patient experiences de qi (obtains qi). A patient's experience of de qi may take on multiple unique manifestations at the needle site itself and/or around the site of needle manipulation including soreness, aching, numbness, tingling, and even warmth. Electrical stimulation of acupuncture needles will be done at the frequency of 15 Hz with continuous wave in order to ensure patient comfort, lasting 30 minutes.

Participants in the above 2 groups will individually be needled for 4 days, beginning on the day before first day of chemotherapy and ending on the last day of it. The intervention will be applied for a third consecutive cycle. Only well-trained acupuncturists with >5 years of clinical experience could conduct acupuncture manipulation.

Subjects in the control group (Group C) will not receive acupuncture treatments. They will receive conventional Western medicine treatment only.

**Figure 1:**
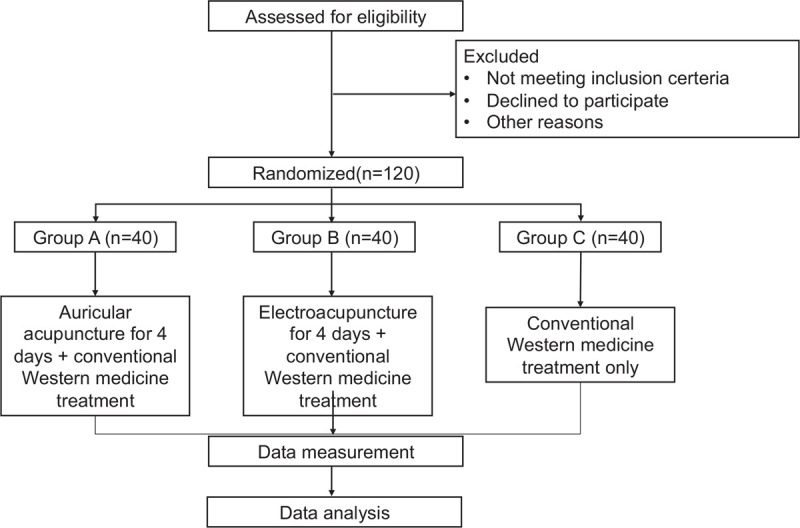
The flow chart of this study.

All patients undergo supervision and evaluation of curative effect at 10 am daily, until discharge. A third party will perform all data analysis. The flow chart of this trial is shown in Fig. 1.

### 2.5 Outcome measures

#### 2.5.1 Primary outcome measure

The primary outcome measure will be the definition and classification of nausea and vomiting (NCI-CTC AE4.0) and the MASCC (Multinational Association for Supportive Care in Cancer) Antiemesis Tool (MAT) score, 24 hours and fourth day after chemotherapy, for a third consecutive cisplatin-based chemotherapy cycle. These 2 scales are used to describe the condition and degree of CINV in cancers, and strongly recommended by different international guidelines and widely applied. They are easy-to-use and evaluate the likelihood of a patient's condition. The definition and classification of nausea and vomiting stages the nausea and vomiting into 3 and 4 grades, respectively; and MAT is comprised of 2 sections: 24 hours later and 4 days later. In terms of MAT, each section has 4 identical items including occurrence of nausea and vomiting, frequency of vomiting, and level of nausea.

#### 2.5.2 Secondary outcome measures

Secondary outcome measures are as follows:

a. Abdominal distension is defined as a score of Likert-type scale at 24 hours and fourth day after chemotherapy. Scores on Likert-type scale range from 1 to 5, with a score of 5 indicating no symptoms; a score of 1 indicating severe distension, interfering sleep (Table 2).
b. The time of first defecation is recorded as a relatively objective indicator to describe the digestive function of participants.
c. The World Health Organization Quality of Life scale will be performed on each patient once a month, 3 times in total.

**Table 2:** The Likert-type scale.

Scores	Symptoms
1	Severe distension, even interfering sleep
2	Salient distension and refuse to feed
3	Endurable distension
4	Slight distension
5	No symptom

#### 2.5.3 Safety evaluation

Any adverse events or abnormalities will be recorded on case report forms no matter what intervention is used. The severity of such adverse events will be described as mild, moderate, or severe, and the relation of the events to the intervention will be evaluated as not related, possibly related, or related. If any serious adverse events occur as the result of a certain intervention, that intervention will be stopped immediately and appropriate corrective action will be taken. Any serious adverse events will be reported promptly to the institutional review board, according to the protocol.

### 2.6 Sample size calculation

Using the frequency of onset as the primary outcome measurement, NQuery Advisor software (version 4.0, Statistical Solutions, Ireland) was used to calculate the appropriate sample size. Based on literature research, the sample size was estimated according to the calculation formula of non-inferiority test. α = 0.025, β = 0.2, non-inferiority margin δ = 15%, ε is the actual difference among groups. One-way test was performed, and the sample size was calculated to show that 103 samples are needed in this study. One hundred eighteen cases were needed in the 3 groups, assuming a 10% non-adherence to treatment and a 5% loss to follow-up. In this study, a total of 120 cases were selected from 3 groups, 40 cases in each group.

### 2.7 Randomization and blinding

Randomization of subjects to 2 groups will be on the basis of a concealed allocation approach using statistical analysis PROCPLAN statements (SAS 9.4; World Headquarters SAS Institute Inc., Cary, NC). A computer-generated list of random numbers will be used to determine the allocation of the participants, with numbered opaque sealed envelopes containing the randomization schedule. The envelopes will be kept by an investigator who is not an assessor in the study and will be informed of the outcomes at the end of the study. In this trial, participants and acupuncture practitioners cannot be blinded. The researchers recording the outcomes and those making the conclusions will be all blinded to patients’ assessments.

### 2.8 Statistical analysis

All continuous data will be expressed as mean ± standard deviation. For these data, a *t* test will be applied to compare normally distributed data between the 2 groups, while the Wilcoxon rank sum test applied for non-normally distributed data. Categorical variables will be presented as frequency and percentage and analyzed using the chi square (χ2) test or the Mann–Whitney *U* test where appropriate. Values of *P* < .05 (2-tailed) will be considered statistically significant. All the efficacy and safety analyses will be performed using the intent-to-treat population. All statistical analyses will be performed using the Statistical Package for the Social Sciences (version 13.0; SPSS; Chicago, IL). A third person performed blinded estimation and statistics of the curative effect.

## 3 Discussion

Acupoint therapies, including EA and AA, have the characteristics of higher acceptability, limit side effects, and inexpensiveness.^[22]^ And yet, the fast growing piles of studies, especially low to moderate evidences,^[23,24]^ do not give us any firm grasp of reasoned acupuncture regimen for preventing CINV. The shortage of the studies which compare different acupuncture therapies cause that clinicians are unable to determine the therapeutic values of them, which is unavailable for selecting the most appropriate treatment.^[25]^ Rather, this is not the whole story. To our best knowledge, there is no single regimen could completely blocking CINV.^[26]^ Therefore, relative studies should focus on all categories of CINV. Unfortunately, most previous researches paid more attention on acute CINV, while delayed CINV is a more common, severe and hard-to-cure subtype.^[27]^ To meet the clinic needs, we design this trail to compare the effectiveness and safety of EA and AA for CINV induced by cisplatin-based regimens in a strictly devised situation, and develop a standardized alternative therapy for medical staff.

To minimize the bias, stratified block randomization and blinding will be applied, and participants will be avoided any communication with others farthest, shaped by separate treatment time and diagnosis rooms.

Despite its strengths, this study has, nevertheless, an obvious limitation. It is a single-center study, which may limit its generalizability so a future multiple-center large-sample size study will be needed. However, the results of this trail will provide more evidence and will help medical staffs make a better choice for patients.

## Acknowledgments

The authors would like to thank Hexiang Xia for his bolstering our morale up whenever we need. Yang Zhang also should be named for her benevolent help in the section of References. They are extremely appreciative of our families’ understanding and supporting.

## Author contributions

**Conceptualization:** Meng-yue Liu, Xiaodong Peng, Chang-sheng Sun.

**Data curation:** Lai Sung.

**Funding acquisition:** Yan-Sen Liao.

**Investigation:** Yi-fei Jiao.

**Methodology:** Chang-sheng Sun.

**Project administration:** Xiaodong Peng.

**Resources:** Lai Sung, Yan-Sen Liao.

**Supervision:** Yi-fei Jiao.

**Writing – original draft:** Meng-yue Liu.

**Writing – review & editing:** Meng-yue Liu.
